# Association of COVID‐19 mitigation measures with preterm birth and cervical length: A cohort study

**DOI:** 10.1002/pmf2.70236

**Published:** 2026-01-23

**Authors:** Nicole Au, Melody Kang, Claire Mcgannon, Shiva Sridhar, Adya Choudary, Daniel L. Rolnik, Kirsten R. Palmer, Atul Malhotra, Ben W. Mol

**Affiliations:** ^1^ Department of Obstetrics and Gynaecology Monash University Melbourne Victoria Australia; ^2^ Women's and Newborn Monash Health Melbourne Victoria Australia; ^3^ Department of Paediatrics Monash University Melbourne Victoria Australia; ^4^ Department of Obstetrics and Gynaecology Amsterdam University Medical Centres, University of Amsterdam Amsterdam The Netherlands

**Keywords:** cervical length, COVID‐19 mitigation measures, preterm birth

## Abstract

**Background:**

We have previously reported that, in metropolitan Melbourne, mitigation measures implemented to mitigate SARS‐CoV‐2 transmission were associated with a reduced preterm birth (PTB) rate, both in spontaneous and iatrogenic births <34 weeks. Here, we assess the impact of mitigation measures on PTB in relation to mid‐pregnancy cervical length (CL).

**Methods:**

We conducted a comparative cohort study in three maternity hospitals in Melbourne, Australia, between women with a previous PTB <32 weeks who were exposed to COVID‐19 mitigation measures while pregnant (lockdown cohort, estimated conception November 1, 2019 to February 29, 2020) and women who were pregnant before mitigation measures (pre‐lockdown cohort, estimated conception November 1, 2018 to February 28, 2019). In our clinics, women with a previous PTB <32 weeks routinely undergo targeted assessment of CL at around 20 weeks. Within each cohort, we assessed the relationship between CL and perinatal outcomes.

**Results:**

We studied 113 women, of whom 60 were in the lockdown group and 53 in the pre‐lockdown group. Baseline characteristics, including CL measurements taken on average at 20.4 and 20.3 weeks of gestation, respectively, were comparable. Mean CL was 38.4 ± 8.8 mm versus 35.8 ± 9.7 mm (*p* = 0.141). Progesterone usage occurred in 5% (lockdown cohort) versus 23% (pre‐lockdown cohort) of women (*p* = 0.006). After adjusting for age, BMI, smoking status, and parity, the rates of PTB <37 weeks were 20% and 26%, respectively (adjusted odds ratio [aOR], 0.60; 95% confidence interval [CI], 0.27–1.35; *p* = 0.199), while rates of PTB <34 weeks were 5% and 19% (aOR, 0.21; 95% CI, 0.06–0.82; *p* = 0.038). In the pre‐lockdown cohort, CL measurements predicted PTB (aOR, 0.79; 95% CI, 0.65–0.90; *p* = 0.009), but this association was not present in the lockdown cohort (aOR, 1.21; 95% CI, 0.95–1.73; *p* = 0.169).

**Conclusion:**

COVID‐19 mitigation measures were associated with a reduction in PTB. The predictive capacity of CL disappeared during mitigation measures. This effect may be attributed to a reduction of PTB in women with a short CL. While the underlying mechanism remains unclear, these findings suggest a potentially modifiable risk factor in lifestyle in high‐risk women with a short CL, warranting further investigation through larger prospective studies.

## 1 INTRODUCTION

Preterm birth (PTB), defined as birth before 37 weeks of gestation, is common, with almost one in every 10 neonates worldwide being born early [1, 2]. This rate has been static for decades. While survival of premature infants has risen greatly since the 1960s, PTB remains associated with higher rates of neonatal complications, long‐term developmental disabilities, and respiratory disease [3].

Understanding the predictors and mechanisms underlying PTB remains critical for improving pregnancy outcomes and developing targeted preventive strategies. One established predictor of PTB risk is cervical length, ideally measured with transvaginal ultrasound [4]. A short cervix is strongly associated with a higher risk of PTB [5, 6]. However, while cervical length screening has been widely used to identify women at higher risk of PTB, there remains a knowledge gap regarding the underlying factors that influence cervical shortening and how external factors may alter this risk predictor.

During the COVID‐19 pandemic, significant reductions in PTB rates and subsequent neonatal care admissions were observed unexpectedly in Melbourne, Australia, where extensive social mitigation measures were implemented to curb the spread of the novel coronavirus (SARS‐CoV‐2) in 2020. This unexpected observation has also been reported in many other regions globally, sparking interest in understanding potential factors that may have contributed to these reductions [7, 8, 9, 10].

In 2023, our group published a cohort study comparing pregnancy outcomes during COVID‐19 mitigation to those from the previous pre‐pandemic year, and found a strong reduction in PTBs before 34 weeks. This impact was most pronounced in women with a history of PTB [7]. These findings suggest that lifestyle changes during COVID‐19 mitigation measures may have influenced factors related to PTB risk. However, the impact of these changes on cervical length, a key predictor of PTB, has not been explored. Investigating whether COVID‐19 mitigation measure–related behavioral and environmental shifts affected cervical shortening could offer insights into potential mechanisms driving PTB risk, possibly linking it to modifiable factors such as stress, activity levels, or other influences of social restrictions. In this study, we investigated the impact of COVID‐19 mitigation measures on mid‐trimester cervical length in relation to gestational age at birth in women with a previous PTB. By evaluating cervical length in relation to gestational age at birth, we aimed to address gaps in knowledge surrounding modifiable risk factors for PTB and to provide insights for future preventive interventions aimed at reducing PTB rates.

## 2 METHODS

### 2.1 Data collection and entry

We performed a cohort study at Monash Health, Melbourne, Australia. Monash Health comprises of three separate hospitals caring for pregnant women and their children (one tertiary and two primary centers), with over 10,000 births per year. Data were extracted from the birth registry of the three hospitals (Birthing Outcomes System [BOS], version 6.04; Management Consultants and Technology Services).

The BOS registry collects pregnancy details that are recorded during antenatal care or immediately after birth by attending clinicians, and neonatal data are recorded at discharge of the neonate. Additionally, cervical length measurements were obtained from internal radiology results and scanned medical records for external radiology. If multiple cervical lengths were recorded between 17 and 23 weeks, the length collected closest to 20 weeks’ gestation was used.

### 2.2 Study population

We studied singleton pregnancies in women with a previous PTB <32 weeks, either through a spontaneous or an iatrogenic cause. Women whose pregnancy was complicated by a major fetal abnormality (an anatomical or chromosomal abnormality with significant consequences) were excluded from analysis. We registered maternal baseline and pregnancy characteristics, including obstetric history, as well as maternal and perinatal outcomes, including mode of birth and the occurrence of antenatal or neonatal complications such as stillbirth, perinatal death, and major congenital anomaly.

We compared women with a previous PTB <32 weeks who were exposed to mitigation measures (lockdown group) to women with a previous PTB <32 weeks who were pregnant before mitigation measures (pre‐lockdown group). To do so, pregnant women with a previous PTB <32 weeks who had an estimated conception date between 1 November 2019 and 29 February 2020 (lockdown group) or between 1 November 2018 and 28 February 2019 (pre‐lockdown group) and who delivered after 20 weeks' gestation were included. The conception date range for the lockdown cohort was chosen to ensure that pregnancies occurred entirely within the main lockdown period, which lasted 111 consecutive days and ended in October 2020 [11]. This approach ensures that women in the lockdown cohort experienced their pregnancy during a time when mitigation measures were consistently in place. Women in the first group were pregnant during the SARS‐CoV‐2 pandemic and the COVID‐19 mitigation measures in Melbourne to mitigate viral spread and were deemed the exposed group. The control group contained women pregnant during the same season in the year prior, when no mitigation measures were in place.

Melbourne, Victoria underwent six non‐consecutive periods of COVID‐19 mitigation measures from March 30, 2020 to October 2021, accumulating 262 days in lockdown [11]. Measures included daily curfews after 8 pm, mask‐wearing in public, social distancing measures of at least 1.5 m, as well as the implementation of only a few permitted reasons to leave the house—such as for exercise, healthcare, food supplies, and for work or education for 1 hour each day, limited to a 5 km travel zone, and limitation of social gathering including closure of restaurants, shops, and public events. Only “essential” workers were allowed to leave the house for longer.

As part of our routine protocol for women with previous extreme PTB, we measured cervical length around 18–20 weeks by transvaginal approach.

### 2.3 Outcomes and covariates

The main outcomes assessed in this study were gestational age at birth and mid‐trimester cervical length. From the reported gestational age at birth, we derived two dichotomous outcomes: PTB before 34 weeks and PTB before 37 weeks. In our analyses of these outcomes, covariate adjustments were made for maternal age, body mass index (BMI), parity, and smoking status, which have been previously shown to be associated with the risk of PTB.

### 2.4 Statistical analysis

Baseline demographic and clinical characteristics were collected for both the lockdown and pre‐lockdown cohorts. Categorical variables are presented as n (%). Continuous variables are summarised using mean (SD) when approximately normally distributed (e.g. age) and median (IQR) when skewed (e.g. BMI).

We compared the distributions of mean mid‐trimester cervical lengths for both the lockdown and pre‐lockdown cohorts, stratified by gestational age at birth (<28 weeks, 28–33 weeks, 34–37 weeks, and term birth). The reference group for this study is the non‐exposed pre‐lockdown cohort. The association between COVID‐19 mitigation measures and cervical lengths was assessed using linear regression within the lockdown and pre‐lockdown cohorts. We performed both unadjusted and adjusted analyses. The model assumption of normality of residuals was tested using residuals versus fitted plots.

Logistic regression models were utilized to examine the relationship between cervical length and PTB <34 and <37 weeks in both pre‐lockdown and lockdown cohorts, adjusted for age, BMI, current smoking status, parity and cervical length. Estimations are presented as adjusted odds ratios (aORs) along with 95% confidence intervals (CIs). Receiver‐operating characteristics (ROC) analysis was used to evaluate the predictive performance of the unadjusted univariate model. DeLong's test was conducted to compare the area under the ROC curves (AUCs) and determine if there was a statistically significant difference in the discriminatory abilities between the models.

Analyses were performed using R Software version 4.3.2 (R Development Core Team. R Foundation for Statistical Computing) [12].

## 3 RESULTS

**FIGURE 1:**
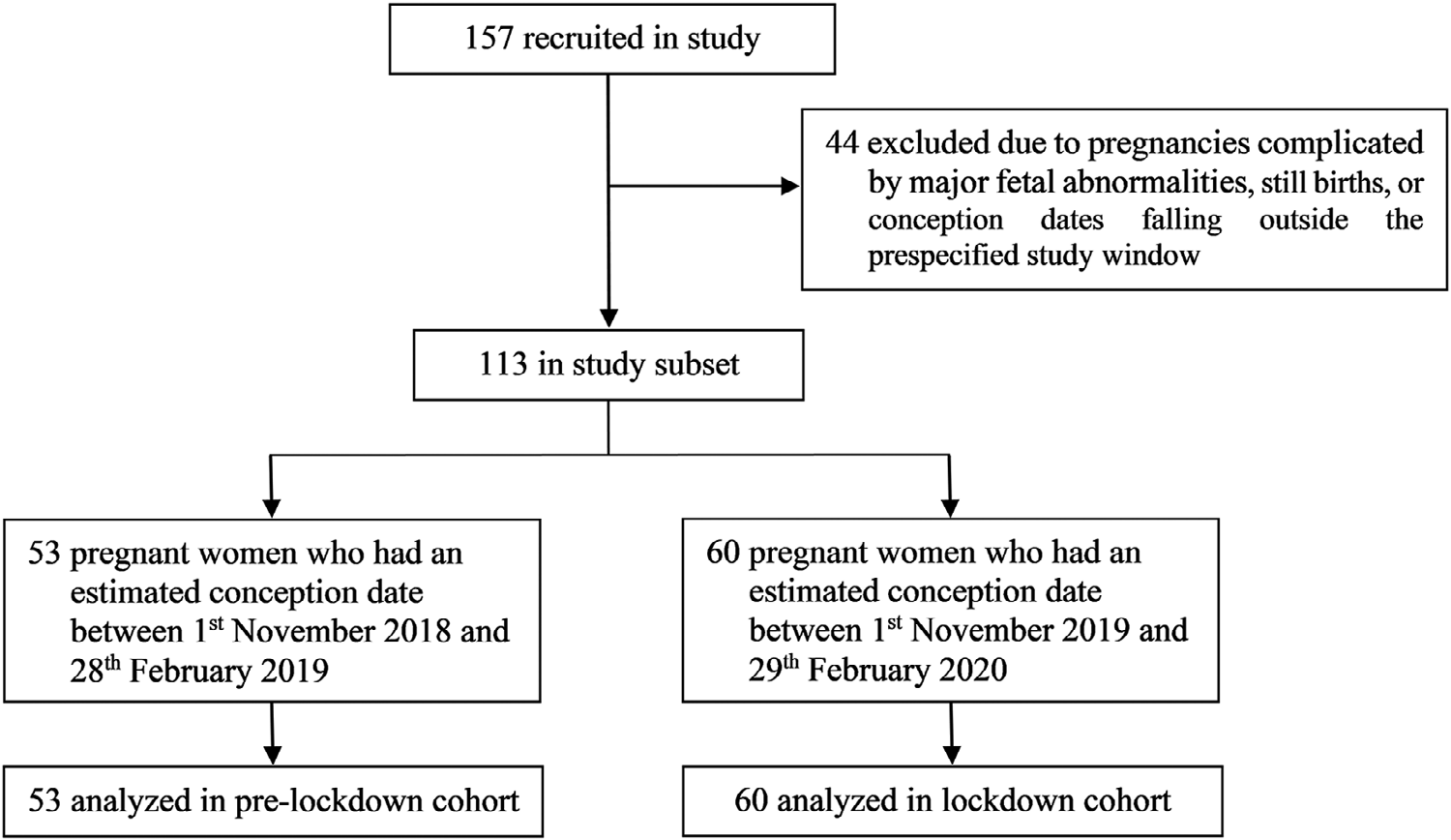
PRISMA flow chart of study design and inclusions.

A total of 157 participants with conception dates of singleton pregnancies between the defined pre‐lockdown and lockdown periods were recruited for the study. Of these, 44 participants (28.1%) were excluded due to pregnancies complicated by major fetal anomalies, still births, or conception dates falling outside the prespecified window. We therefore analyzed data from 113 women, 60 in the lockdown group and 53 in the pre‐lockdown group (Figure 1). Across both groups, there were no statistically significant differences in baseline characteristics (Table 1).

**TABLE 1:** Demographic data and clinical characteristics of study participants.

	COVID‐19 mitigation		
Baseline characteristics	Lockdown *N* = 60^a^	Pre‐lockdown *N* = 53^a^	*p* value^b^
Age (years)	33.1 ± 5.1	32.9 ± 4.2	0.956
BMI (kg/m^2^)—median (IQR)	26.7 (23.2–33.3)	26.7 (23.3–32.2)	0.966
Marital status			0.954
Married/de facto	53 (88)	47 (89)	
Single/Divorced	7 (12)	6 (11)	
Gravidity			0.212
2	10 (17)	16 (30)	
3	14 (23)	12 (23)	
4 or more	36 (60)	25 (47)	
Parity			0.141
1	16 (27)	23 (43)	
2	24 (40)	14 (26)	
3 or more	20 (33)	16 (30)	
Current smoker	8 (13)	5 (9.4)	0.517
Prophylactic progesterone use	3 (5.0)	12 (22.6)	0.006
Region of origin			0.865
Australia/NZ	26 (43)	21 (40)	
Central/South Asia	17 (28)	15 (28)	
Europe/UK	2 (3.3)	2 (3.8)	
Middle East	0 (0)	1 (1.9)	
North/Central America	1 (1.7)	0 (0)	
Pacific Islands	0 (0)	2 (3.8)	
Southeast/East Asia	9 (15)	8 (15)	
Sub‐Saharan Africa	5 (8.3)	4 (7.5)	

Abbreviations: BMI, body mass index; IQR, interquartile range; NZ, New Zealand; UK, United Kingdom.

^a^ Data are mean ± SD for continuous characteristics or *n* (%) for categorical characteristics unless specified.

^b^ Wilcoxon rank sum test; Pearson's Chi‐squared test; Fisher's exact test.

### 3.1 Association between COVID‐19 mitigation measures and cervical lengths

Mean cervical length was comparable between the two groups, measuring 38.4 ± 8.8 mm lockdown (range: 9–63 mm) compared to 35.8 ± 9.7 mm (range: 1.4–61 mm) pre‐lockdown (*p* = 0.131) (Table 2). The rates of PTB <37 weeks were 20% and 26%, respectively (aOR, 0.60; 95% CI, 0.27–1.35; *p* = 0.199), while the rates of PTB <34 weeks were 5% and 19%, respectively (aOR, 0.21; 95% CI, 0.06–0.82; *p* = 0.038) (Table 4).

**TABLE 2:** Association between COVID‐19 mitigation restrictions and cervical length.

	COVID‐19 mitigation		Unadjusted		Adjusted^b^	
Outcome	Lockdown *N* = 60^a^	Pre‐lockdown *N* = 53^a^	Mean differences^a^	*p* value	Mean differences^a^	*p* value
Cervical length (mm)	38.4 ± 8.8	35.8 ± 9.7	2.58 (‐0.87 to 6.04)	0.141	2.68 (‐0.81to 6.17)	0.131
Gestational age at measurements (weeks)	19.9 ± 1.4	20.5 ± 0.9				

^a^ Coefficients are mean differences estimated by linear regression with cervical length as the outcome and COVID‐19 mitigation group as the predictor.

^b^ Adjusted for age, body mass index, smoking, and parity.

### 3.2 Association between cervical lengths and PTBs

In the lockdown cohort, cervical length was not a significant predictor for both PTB <34 weeks; aOR, 1.21 (95% CI, 0.95–1.73; *p* = 0.169) and PTB <37 weeks; aOR, 1.05 (95% CI, 0.95–1.18; *p* = 0.403) (Table 3). Whereas in the pre‐lockdown cohort, cervical length was a significant predictor for PTB both prior to 34 weeks (aOR, 0.79; 95% CI. 0.65–0.90; *p* = 0.003) and 37 weeks (aOR, 0.88; 95% CI, 0.79–0.96; *p* = 0.009).

**TABLE 3:** Logistic regression analysis of preterm birth rates using cervical length as a predictor in pre‐lockdown and lockdown cohorts, occurring at <37 and <34 weeks.

	COVID‐19 mitigation									
	Lockdown *N* = 60					Pre‐lockdown *N* = 53				
		Unadjusted		Adjusted^2^			Unadjusted		Adjusted^2^	
Outcome	Number of PTB cases	Odds ratio	*p* value^a^	Odds ratio	*p* value^a^	Number of PTB cases	Odds ratio	*p* value^a^	Odds ratio	*p* value^a^
Preterm <37 weeks	12 (20%)	1.04 (0.97–1.13)	0.319	1.05 (0.95–1.18)	0.403	14 (26%)	0.91 (0.83–0.98)	0.018	0.88 (0.79–0.96)	0.009
Preterm <34 weeks	3 (5.0%)	1.07 (0.93–1.24)	0.346	1.21 (0.95–1.73)	0.169	10 (19%)	0.87 (0.77–0.95)	0.006	0.79 (0.65–0.90)	0.003

^a^ Wald *z*‐tests.

^b^ Adjusted for age, body mass index, current smoking status, and parity.

**FIGURE 2:**
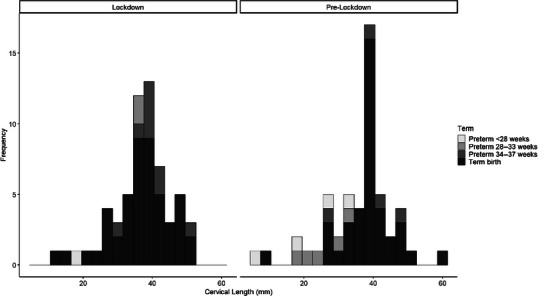
Histogram of cervcial lengths versus number of PTBs occuring <34 weeks, <37 weeks, and term births, in lockdown versus pre‐lockdown cohorts.

Figure 2 compares cervical length in pre‐lockdown and lockdown periods. In pre‐lockdown, shorter cervical lengths were associated with increased risk of PTB; however, this association disappeared during COVID‐19 mitigation measures, with most PTBs occurring in women with cervical lengths ≥ 40 mm.

**FIGURE 3:**
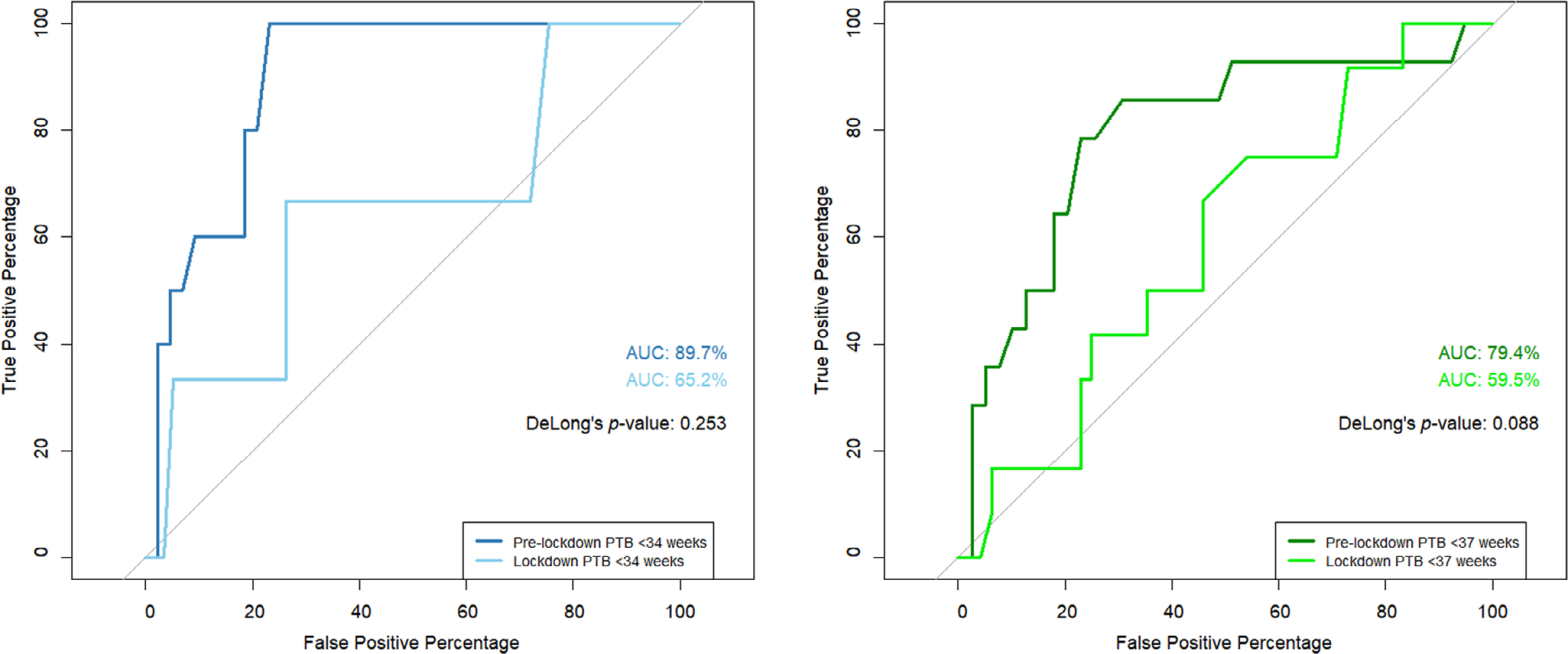
Receiver‐operating characteristics (ROC) curves comparing univariate logistic regression models between lockdown cohorts using cervical length as the predictor: (1) Preterm births <34 weeks, (2) Preterm births <37 weeks

Figure 3 shows ROC curves for PTB prediction using univariate logistic regression models. AUCs were numerically higher in the pre‐lockdown models (AUC 89.7% for <34 weeks and 79.4% for <37 weeks) than in the lockdown models (AUCs 65.2% and 59.5%); however, DeLong's Tests showed no statistically significant difference in AUCs between periods (*p* = 0.253 and *p* = 0.088, respectively), suggesting comparable predictive performance of both models.

### 3.3 Analysis of spontaneous and iatrogenic PTBs

At <37 weeks, the aOR of 0.24 (95% CI, 0.07–0.77; *p* = 0.017) indicates a 76% lower risk of spontaneous PTB in the lockdown cohort compared to pre‐lockdown. For <34 weeks, the OR of 0.11 (95% CI, 0.01–0.88; *p* = 0.038) reflects an 89% risk reduction. These findings suggest a more pronounced risk reduction for earlier spontaneous PTBs during COVID‐19 mitigation, potentially due to beneficial or mitigating factors. However, the risk of spontaneous PTB increases between 34 and 37 weeks (Table 4).

**TABLE 4:** Association between COVID‐19 mitigation restrictions and preterm births, stratified by type of preterm birth.

	COVID‐19 mitigation exposure		Unadjusted odds ratio		Adjusted odds ratio^b^	
Outcome	Lockdown *N* = 60^a^	Pre‐lockdown *N* = 53^a^	Coefficient	*p* value	Coefficient	*p* value
Preterm birth <37 weeks	12 (20%)	14 (26%)	0.67 (0.31–1.45)	0.313	0.60 (0.27–1.35)	0.199
Spontaneous	4 (6.7%)	12 (23%)	0.27 (0.09–0.82)	0.022	0.24 (0.07–0.77)	0.017
Iatrogenic	8 (13%)	2 (3.8%)	3.04 (0.65–14.32)	0.160	2.87 (0.57–14.55)	0.203
Preterm birth <34 weeks	3 (5.0%)	10 (19%)	0.25 (0.07–0.90)	0.034	0.21 (0.06–0.82)	0.024
Spontaneous	1 (1.7%)	8 (15%)	0.11 (0.01–0.84)	0.034	0.11 (0.01–0.88)	0.038
Iatrogenic	2 (3.3%)	2 (3.8%)	0.78 (0.11–5.58)	0.810	0.46 (0.05–4.15)	0.487

^a^ Data are *n* (%) for categorical characteristics using logistic regression.

^b^ Adjusted for age, body mass index, current smoking status, parity, and cervical length.

In the lockdown cohort, there were eight cases of iatrogenic PTBs in the <37 weeks group, but fewer spontaneous PTBs. In contrast, the pre‐lockdown cohort only recorded two iatrogenic PTBs in both the <34 weeks and <37 weeks groups, which is a quarter of the cases reported in the lockdown cohort. The results of the Schoenfeld residuals test support the validity of the proportional hazards assumption.

### 3.4 Post hoc analysis to test for confounding of prophylactic progesterone use

Because prophylactic progesterone use differed significantly between the two cohorts, we conducted a post hoc analysis to assess its potential confounding effect by including it as a covariate in the logistic regression model for PTB. We compared the effect estimates from an unadjusted model with those from a model adjusted for prophylactic progesterone use (Table 3). Prophylactic progesterone use did not confound the association between cervical length and PTB, as the effect estimate for cervical length remained unchanged after adjustment (Table S1). Furthermore, prophylactic progesterone use was not independently associated with PTB (*p* > 0.05).

## 4 DISCUSSION

This study suggests a reduction in PTB during COVID‐19 mitigation measures. The finding of an association between shorter cervical lengths and increased PTB in the pre‐lockdown group, but not in the lockdown group, may indicate that cervical length was no longer a predictor of PTB during the COVID‐19 mitigation period. Although the underlying mechanism of action remains uncertain, one hypothesis is that the impact of lockdown measures may have led to fewer short cervices and therefore lower rates of spontaneous PTB, thereby diminishing the predictive capacity of cervical length during the lockdown period. However, we acknowledge a limitation given by the small sample size of our study, where the rare PTB incidences in the lockdown group could be a potential explanation why cervical length is not predicting PTB in this group. We also note the potential for selection bias in the participants included in this study. The spread of COVID‐19 and the rollout of the vaccination program in Australia may have influenced pregnancy planning decisions. Some individuals may have delayed conception due to concerns about COVID‐19 infection during pregnancy or uncertainty surrounding vaccination, potentially altering the profile of those who conceived during the lockdown period. In addition, during COVID‐19 mitigation measures, some mothers may have avoided hospitals or routine antenatal visits due to fear of infection. Both of these hypothetical factors may suggest that women with higher health consciousness were more mindful during pregnancy or more likely to have planned their pregnancies during this period. This could result in selection bias, with the lockdown cohort comprising women who generally had healthier lifestyles and therefore a lower baseline risk.

Although not statistically significant, iatrogenic PTBs were found to increase between 34 and 37 weeks’ gestation in the lockdown group, particularly in the late preterm period, despite fewer spontaneous PTBs. Such an increase in iatrogenic PTBs during COVID‐19 mitigation measures may be due to changes in clinical practice during lockdown, differences in case mix, or random variation given the small number of events. However, we acknowledge that the small sample size limitation also applies to this stratified analysis, the non‐significant findings for iatrogenic PTB may reflect insufficient power rather than a true absence of association as depicted by the odds ratio now. Despite this limitation, we believe the stratified analysis provides exploratory insights into potential differential effects of lockdown measures on spontaneous versus iatrogenic PTB, which may have distinct etiologies. These findings should be interpreted with caution and warrant validation in larger cohorts. Our prior work found no evidence that changes in antenatal‐care delivery during COVID‐19 mitigation impacted the detection of risk factors during pregnancy, such as pre‐eclampsia and fetal growth restriction, which may otherwise have been mitigated or monitored more carefully during the pre‐lockdown period [13, 14]. Consistent with these findings, our other recent study observed fewer spontaneous preterm labors but higher rates of preterm prelabor rupture of membranes, a pattern that may otherwise have contributed to iatrogenic PTB in the absence of spontaneous preterm labor [15]. Another hypothesis is that the women who did not have an early spontaneous PTB due to mitigation measures, but remained pregnant for longer, had a higher probability of pregnancy complications [16].

The exact mechanism through which COVID‐19 mitigation measures reduce PTB is unknown. It is possible that during mitigation measures, women were exposed to fewer transmitted infections, which may have a role in the mechanism of PTB [8]. Improved air quality during COVID‐19 mitigation measures may also have contributed beneficially to a reduced PTB rate [9]. Major changes in behavior, such as a reduction in high‐risk activities and exercise, as well as increased restful behavior due to the nature of mitigation measures, are in our opinion the most likely explanation [17, 18]. Under that hypothetical mechanism, we have started a pilot feasibility randomized trial that mimics COVID‐19 measures in a lifestyle intervention [19].

Although current evidence is insufficient to confirm a causal relationship, and the underlying mechanism also remains unclear, these findings point to a potential modifiable risk factor and support further exploration into behavioral and environmental interventions aimed at preventing PTB. Further research is needed to clarify any potential association. Larger prospective studies are needed to confirm these findings and to explore whether lifestyle changes similar to those seen during the pandemic could help reduce PTB in high‐risk women.

A limitation of our study was the small sample size of 60 versus 53 individuals in the lockdown and pre‐lockdown cohorts, respectively, due to the narrowly defined cohort (prior PTB <32 weeks) and the short ascertainment window corresponding to the period of pronounced lockdown. However, these are the only numbers attainable under such historically unique mitigation conditions, which are unlikely to be replicated in future natural settings. This can result in lower statistical power and higher degree of uncertainty with wider CIs, which in turn increases the uncertainty of our estimates. Expanding the scope through further studies and data analysis would allow a broader examination of the effects of COVID‐19 mitigation measures on these and other known contributing factors to PTBs. Although COVID‐19 mitigation measures did not result in a measurable difference in mean cervical length between the lockdown and pre‐lockdown cohorts, cervical length itself was a significant predictor of PTB in the pre‐lockdown cohort. This suggests that while cervical shortening is an important risk factor for PTB, it does not appear to be the causal pathway through which mitigation measures exerted their effect in this study. Instead, mitigation measures could theoretically influence pathways related to cervical shortening. Future studies assessing a potential preventative effect of mitigation measures could therefore be limited to women with a cervical length below 25 mm, where any influence on cervical shortening would be most detectable.

## 5 CONCLUSION

This study provides further evidence that COVID‐19 mitigation measures were associated with a reduction in PTBs, particularly among women with a short mid‐trimester cervical length. PTB before 34 weeks occurred less often during lockdown, while rates before 37 weeks were similar. Notably, cervical length was no longer a significant predictor of PTB during the lockdown period, suggesting that COVID‐19 mitigation measures may have directly or indirectly influenced the physiological processes involved in cervical shortening. Despite this change in association, the overall ability of cervical length–based models to predict PTB was comparable between periods.

## CONFLICT OF INTEREST STATEMENT

N.A. is supported by a Research Training Stipend, provided by the Australian Government. D.L.R., A.M., K.R.P., and B.W.M. are supported by NHMRC Investigator grants (GNT2025670, GNT2008793, GNT2009765, and GNT1176437, respectively); B.W.M. reports consultancy, travel support, and research funding from Merck and consultancy for Ferring, Organon, UNILAB, and Norgine.

## FUNDING INFORMATION

The authors received no specific funding for this work.

## ETHICS STATEMENT

Ethical approval for this study was obtained from the Monash Health Human Research Ethics Committee (approval number QA/69113/MonH‐2020‐235157).

## Supporting information



Supplementary Information

## References

[1] Goldenberg, R. L. , J. F. Culhane , J. D. Iams , and R. Romero . 2008. “Epidemiology and Causes of Preterm Birth.” Lancet 371(9606): 75–84. 18177778 10.1016/S0140-6736(08)60074-4 PMC7134569

[2] Victoria State Government . 2014. Premature Babies. State of Victoria: Victoria State Government. Updated August 2014. Available From: https://www.betterhealth.vic.gov.au/health/healthyliving/premature‐babies.

[3] “Institute of Medicine (US) Roundtable on Environmental Health Sciences, Research, and Medicine.” The Role of Environmental Hazards in Premature Birth: Workshop Summary, edited by D. R. Mattison , et al., 2003. Washington, DC: National Academies Press (US). 10.17226/10842. 25009846

[4] Coutinho, C. M. , A. Sotiriadis , A. Odibo , A. Khalil , F. D'Antonio , H. Feltovich , L. J. Salomon , et al. 2022. “ISUOG Practice Guidelines: Role of Ultrasound in the Prediction of Spontaneous Preterm Birth.” Ultrasound in Obstetrics & Gynecology 60(3): 435–56. 35904371 10.1002/uog.26020

[5] Iams, J. D. , R. L. Goldenberg , P. J. Meis , B. M. Mercer , A. Moawad , A. Das , E. Thom , et al. 1996. “The Length of the Cervix and the Risk of Spontaneous Premature Delivery. National Institute of Child Health and Human Development Maternal Fetal Medicine Unit Network.” New England Journal of Medicine 334(9): 567–72. 8569824 10.1056/NEJM199602293340904

[6] Berghella, V. , and A. D. Mackeen . 2011. “Cervical Length Screening With Ultrasound‐indicated Cerclage Compared With History‐indicated Cerclage for Prevention of Preterm Birth: A Meta‐Analysis.” Obstetrics and Gynecology 118(1): 148–55. 21691173 10.1097/AOG.0b013e31821fd5b0

[7] Rolnik, D. L. , A. Matheson , Y. Liu , S. Chu , C. Mcgannon , B. Mulcahy , A. Malhotra , K. R. Palmer , R. J. Hodges , and B. W. Mol , 2021. “Impact of COVID‐19 Pandemic Restrictions on Pregnancy Duration and Outcome in Melbourne, Australia.” Ultrasound in Obstetrics & Gynecology 58(5): 677–87. 34309931 10.1002/uog.23743

[8] Matheson, A. , C. J. McGannon , A. Malhotra , K. R. Palmer , A. E. Stewart , E. M. Wallace , B. W. Mol , R. J. Hodges , and D. L. Rolnik , 2021. “Prematurity Rates During the Coronavirus Disease 2019 (COVID‐19) Pandemic Lockdown in Melbourne, Australia.” Obstetrics and Gynecology 137(3): 405–7. 33543904 10.1097/AOG.0000000000004236 PMC7884082

[9] Jasper, B. , T. Stillerova , C. Anstey , and E. Weaver . 2022. “Reduction in Preterm Birth Rates During and After the COVID‐19 Lockdown in Queensland Australia.” Australian and New Zealand Journal of Obstetrics and Gynaecology 62(6): 851–8. 35581948 10.1111/ajo.13538 PMC9348165

[10] Calvert, C. , M. Brockway , H. Zoega , J. E. Miller , J. V. Been , A. K. Amegah , A. Racine‐Poon , et al. 2023. “Changes in Preterm Birth and Stillbirth During COVID‐19 Lockdowns in 26 Countries.” Nature Human Behaviour 7(4): 529–44. 10.1038/s41562-023-01522-y PMC10129868 36849590

[11] Dunstan, J. “Melbourne Marks 200 Days of COVID‐19 Lockdowns Since the Pandemic Began.” *ABC News*, August 18, 2021.

[12] R Development Core Team . 2022. R: A Language and Environment for Statistical Computing. 4.3.2 ed. Vienna, Austria: R Foundation for Statistical Computing.

[13] Palmer, K. R. , M. Tanner , M. Davies‐Tuck , A. Rindt , K. Papacostas , M. L. Giles , K. Brown , et al. 2021. “Widespread Implementation of a Low‐Cost Telehealth Service in the Delivery of Antenatal Care During the COVID‐19 Pandemic: An Interrupted Time‐Series Analysis.” Lancet 398(10294): 41–52. 34217399 10.1016/S0140-6736(21)00668-1 PMC8248925

[14] Thirugnanasundralingam, K. , M. Davies‐Tuck , D. L. Rolnik , M. Reddy , B. W. Mol , R. Hodges , and K. R. Palmer , 2023. “Effect of Telehealth‐Integrated Antenatal Care on Pregnancy Outcomes in Australia: An Interrupted Time‐Series Analysis.” Lancet Digital Health 5(11): e798–811. 37890903 10.1016/S2589-7500(23)00151-6

[15] Liu, Y. A. , A. Matheson , R. Sleaby , B. Mulcahy , K. R. Palmer , R. J. Hodges , B. W. Mol , A. Malhotra , and D. L. Rolnik , 2025. “Characteristics of Preterm Births During COVID‐19 Mitigation Measures.” Australian and New Zealand Journal of Obstetrics and Gynaecology 65(1): 114–20. 38943364 10.1111/ajo.13853

[16] Latif, S. , and C. Aiken . 2021. “Prolonged Pregnancy.” Obstetrics, Gynaecology & Reproductive Medicine 31(6): 170–4.

[17] Stockwell, S. , M. Trott , M. Tully , J. Shin , Y. Barnett , L. Butler , D. McDermott , F. Schuch , and L. Smith , 2021. “Changes in Physical Activity and Sedentary Behaviours From Before to During the COVID‐19 Pandemic Lockdown: A Systematic Review.” BMJ Open Sport & Exercise Medicine 7(1): e000960. 10.1136/bmjsem-2020-000960 PMC7852071 34192010

[18] Zhang, J. , Y. Xiao , S. Bai , S. Lin , S. Du , and Z. Wang . 2024. “The Association Between Exercise During Pregnancy and the Risk of Preterm Birth.” International Journal of Women's Health 16: 219–28. 10.2147/IJWH.S447270 PMC10854229 38344255

[19] Sridhar, S. , B. W. Mol , R. Hodges , K. R. Palmer , S. Sundram , R. de Carvalho Pacagnella , R. T. Souza , et al. 2023. “Feasibility of a Pregnancy Intervention Mimicking Viral Transmission Mitigation Measures on the Incidence of Preterm Birth in High‐Risk Pregnant Women Enrolled in Antenatal Clinics in Melbourne, Australia: Protocol for a Pilot, Randomised Trial.” BMJ Open 13(12): e075703. 10.1136/bmjopen-2023-075703 PMC10759123 38154903

