# Supplementary material for: Association of COVID‐19 mitigation measures with preterm birth and cervical length: A cohort study
Source: Pregnancy (Hoboken). 2026 Jan 23;2(1):e70236. doi: 10.1002/pmf2.70236 (PMC13344763; doi:10.1002/pmf2.70236)
Supplement: Supplementary file 1 [file PMF2-2-e70236-s001.docx]

**Supplementary table 1: Post-hoc confounding analysis of prophylactic progesterone use in logistic regression models of preterm birth (<37 and <34 weeks) using cervical length as the predictor in pre-lockdown and lockdown cohorts**

| **Outcome** | **COVID-19 mitigation** | | | |
| --- | --- | --- | --- | --- |
|  | **Lockdown** N = 60 | | **Pre-lockdown** N = 53 | |
|  | **Odds Ratio** | **p-value^1^** | **Odds Ratio** | **p-value^1^** |
| **Preterm <37 weeks** | 1.03 (0.95 to 1.12) | 0.485 | 0.92 (0.84 to 0.99) | 0.033 |
| **Preterm <34 weeks** | 1.05 (0.91 to 1.24) | 0.528 | 0.89 (0.79 to 0.96) | 0.013 |
| *^1^* Wald z-tests | | | | |
